# Using the snow-day fraction to measure climatic change in southern Ontario (Canada): historical trends in winter season precipitation phase

**DOI:** 10.1007/s00704-022-04267-2

**Published:** 2022-11-07

**Authors:** Micah J. Hewer, William A. Gough

**Affiliations:** Department of Physical and Environmental Sciences, University of Toronto at Scarborough, 1265 Military Trail, Toronto, ON M1C 1A4 Canada

## Abstract

Global temperatures are increasing, and regional precipitation patterns are changing. Snow is an excellent indicator of regional climate change; 50 years of temperature and precipitation data were analysed from weather stations located within the five most populated cities of Ontario (Canada). Recorded measurements for temperature and precipitation were converted into binary values to indicate the frequency of rain days, snow days, wet days (when total precipitation is greater than 0.2 mm) and freezing days (when the average temperature is less than 0 °C); then, these values were summed over each winter season from 1970/71 to 2019/20. The snow-day fraction was calculated from the seasonal totals by dividing the total number of snow days by the total number of wet days. Historical trends were detected using Pearson’s *R*, Kendall’s Tau and Spearman’s Rho. Differences in mean values between the first decade (1971–1980) and the last decade (2011–2020) within the time series for the snow-day fraction and total freezing days were determined using Student’s *t*-tests. During the winter season in southern Ontario (December 1 to March 31), total snow days, total wet days, the snow-day fraction and freezing days were all decreasing at statistically significant rates (90 to 99% confidence levels) across four of the five cities studied (Toronto, Ottawa, Hamilton and London). Mississauga was the exception, being the only city where rain days were increasing, but no trends were detected for snow days or wet days. The snow-day fraction was decreasing in Mississauga but not at a statistically significant rate, despite freezing days decreasing at the greatest rate compared to the other four cities. Total freezing days were highly correlated with the snow-day fraction during the winter season, being able to explain 61 to 76 percent of the observed variability, where Mississauga recorded the weakest correlation and London recorded the strongest correlation.

## Introduction

The Earth’s surface has been warming since the industrial revolution, and the climates human experience worldwide have been changing. The Intergovernmental Panel on Climate Change (IPCC 2014) states that observed changes in the Earth’s climate are unprecedented over recent millennia. Mean global surface air temperatures (temperatures above both land and ocean surfaces) have increased by 0.87 °C when comparing 1850 − 1900 to 2006 − 2015 (IPCC 2019). Land surface air temperatures have risen even faster than global surface air temperatures, warming by 1.53 °C between this preindustrial period and the recent decade. Global warming has been linked to climatic changes over land surfaces: such as increased heatwaves and droughts, increased rainfall and flooding, decreased snowfall and melting ice, and changing precipitation patterns and shifting climate zones (IPCC 2019). Rising temperatures have also been linked to climate-induced environmental changes, such as melting glaciers and reduced ice coverage, coastal erosion and flooding, and biodiversity loss and species extinction, while also threatening human health and food security (IPCC 2018).

Canada’s climate has been warming faster than the average global rate. Zhang et al. (2019) reported an average warming across the country of 1.7 °C from 1948 to 2016, with a warming of 3.3 °C during the winter months. There have been fewer cold nights, cold days and frost days, with conversely more warm nights, warm days and summer days across the country (Vincent and Mekis 2006). Trends show fewer days with extremely low temperatures and more days with extremely high temperatures (Bonsal et al. 2001). Growing seasons have been lengthened (Qian et al. 2010), growing degree days have increased (Bonsal et al. 2001), and fewer freeze–thaw days have been reported (Ho and Gough, 2006; Yagouti et al. 2008).

Precipitation totals have increased across Canada during all four seasons; however, there have been widespread decreases in the amount of precipitation falling as snow across the southern regions of Canada (Vincent et al. 2015). Furthermore, spring precipitation has been shifting from snow to rain across Canada, while the duration of snow cover has been decreasing (Brown et al. 2010; Mekis and Vincent 2011). More days with precipitation and a decrease in consecutive dry days have also been observed across the country (Vincent and Mekis, 2006). Shorter snow-cover seasons, mainly in response to earlier snowmelt, resulting from warmer spring temperatures, have also been reported (Brown et al. 2010; Mudryk et al. 2018). Vincent et al. (2018) found that the number of days with rainfall has been increasing, while the number of days with snowfall has been decreasing across Canada from 1948 to 2012. Similarly, the number of days with heavy rainfall was increasing, while days with heavy snowfall were decreasing (Vincent et al. 2018).

The Canadian province of Ontario has been warming at a slower rate than the national average. Zhang et al. (2000) found that annual mean temperatures increased by 0.5 to 1.5 °C in southern Ontario from 1900 to 1998, with the greatest warming occurring in the winter season. Zhang et al. (2019) reported that average annual temperatures in Ontario have increased by 1.3 °C from 1948 to 2016, while winter months (December − February) warmed by an average of 2.0 °C. Although precipitation amounts have been increasing across Canada, this trend is even more pronounced in southern Ontario (Mekis and Vincent 2011). Decreasing snowfall and increasing rainfall amounts observed across the country were also more pronounced in southern regions of Ontario (Vincent et al. 2018).

Snow as a climate variable is a useful index of climatic variability and change (McCabe et al. 2018). Because snow is climatically sensitive and global temperatures have been increasing, studies have examined changes and trends in snow cover (McCabe and Wolock 2010), snowpack accumulations (McCabe and Wolock 2009), and snowmelt runoff (McCabe and Clark, 2005). Several studies focused on the USA found declining amounts of snow being mainly attributed to increasing temperatures (Hamlet et al. 2005; Mote et al. 2005; Clow 2010). Other studies have explored changes and trends in the fraction of precipitation that has been falling as snow (Karl et al. 1993; Huntington et al. 2004; Knowles et al. 2006; Feng and Hu, 2007; McCabe et al. 2018; Hewer and Gough 2020; Gough 2021), with consistent findings showing that the snowfall to total precipitation fraction has been decreasing across North America.

Several measurements exist for quantifying snow. Snowfall is the amount of precipitation that is intercepted at the Earth’s surface in the form of snow (Groisman and Easterling, 1994; Ueda et al. 2015). Snow cover is the spatial extent of the snow that remains as ground cover (Farmer et al. 2009). Snow depth is a vertical measurement of snow on the ground (Hong and Ye, 2014). Several researchers have employed a snow-to-rain ratio to quantify climatic changes in the winter season precipitation phase (Lapp et al. 2005; Wipf et al. 2009; Berghuijs et al. 2014; Huang et al. 2018; Dong et al. 2019; Hamlet et al. 2020; Hewer and Gough 2020; Gough 2021). Others have studied the fraction of total precipitation that falls as snow to understand the impact of regional warming on the precipitation phase in North America (Karl et al. 1993; Huntington et al. 2004; Knowles et al. 2006; Feng and Hu 2007; McCabe et al. 2018; Hewer and Gough 2020). An increasingly popular approach to studying climatic changes in the precipitation phase considers frequency rather than volume (Vincent et al. 2018), quantifying changes in the fraction of wet days that recorded snow (McAfee et al. 2014; Hewer and Gough 2020), hereafter referred to as the snow day fraction (SDF).

There have been concerns reported with comparing snowfall to total precipitation since catch efficiency is typically lower for snow than for rain (Yang et al. 1998; Groisman and Legates 1994). Gauged precipitation is known to have differing biases for liquid and solid precipitation (Groisman & Easterling, 1994; Rasmussen et al. 2012). Errors in board-measured snowfall can also be substantial, and it is difficult to reliably convert snowfall to its water equivalent (McAfee et al. 2014). To avoid potential errors associated with solid precipitation measurement, McAfee et al. (2014) used SDF to quantify climatic changes in Alaska rather than using the ratio between the snowfall water equivalent and the amount of total precipitation. Hewer and Gough (2020) analysed changes in the snowfall fraction of Huntington et al. (2004), the snow day fraction of McAfee et al. (2014), and a novel rain-to-snow ratio for Toronto (Ontario, Canada) from 1848 to 2017, where all three metrics demonstrated a shift from rain to snow during the winter season; however, they concluded that SDF resulted in the most pronounced time series trend and had the strongest correlation with rising temperatures.

Precipitation and precipitation phase are difficult climate variables to model for future climate change projections (McAfee et al. 2014). Studies that quantify changes in precipitation and precipitation phase have important implications for understanding regional climate change and modelling future climates (Hewer and Gough 2020). Changes in the winter season precipitation phase have important implications for the volume and persistence of snowpacks (Knowles and Cayan 2004; Stewart et al. 2004). Less snowfall and more rainfall also have important implications for snowmelt, runoff and streamflow (Cayan et al. 2001; Stewart et al. 2005). Ongoing changes in the precipitation phase can also affect the timing of spring runoff, river ice-out times and late-winter snow density (Huntington et al. 2004). The combined effects of declining snowfall, snowpacks, snowmelt and run-off also have important implications for agriculture (Hewer and Brunette 2020; Beech and Hewer 2021).

The goal of this study is to quantify historical changes in the winter season precipitation phase across the most populated cities in southern Ontario (Canada). This goal will be guided by the following research objectives:

1. Access 50 years of historical climate data from weather stations located in Ontario’s most populated cities.
2. Convert recorded temperature and precipitation values into daily frequencies of rain, snow and freezing surface temperatures.
3. Calculate historical trends in rain days, snow days, freezing days and the snow day fraction.
4. Correlate changes in the frequency of the winter season precipitation phase with average surface temperatures and total freezing days.
5. Explore differences between the five cities in southern Ontario.

The intended outcome of this study is to quantify historical climatic changes at regional and local scales while also capturing aspects of lived experience with environmental change.

Climate change is difficult to comprehend from a human perspective because it is such a large phenomenon over both space and time (Moloney et al. 2014; Milkoreit 2015; Boulton 2016). Snow is a useful variable for detecting climatic variability and change (McCabe and Wolock 2010; McCabe et al. 2018), since shifting the precipitation phase from snow to rain is more discernible from a human perspective than rising temperatures alone. The snow day fraction helps capture climate change from a lived experience perspective (Renouf 2021; Abbott and Wilson 2014), as the frequency of days with either rain or snow over time is more recognisable from a human perspective than the quantity of rain or snow over time. The basis for this assumption is that people struggle to conceptualize the quantity of snow or rain over a 24-h period but are more cognitively aware of whether it rained or snowed on that day (excluding days with mixed precipitation). Pahl et al. (2014) suggest that humans living in urban environments face even greater barriers to recognising climatic changes. This study also contributes to population geographies of climate change (Bailey 2011), considering the most populated cities across southern Ontario and illustrating how these human populations have experienced climatic changes in the winter season precipitation phase over their lifetimes (50 years).

Using the snow-day fraction to quantify climatic changes in the winter season precipitation phase overcomes measurement errors associated with solid forms of precipitation (Yang et al. 1998; Groisman and Legates 1994; Groisman and Easterling 1994; Rasmussen et al. 2012). Using the frequency-based snow-day fraction may therefore be a more accurate measure of climatic change than the volume-based measure of comparing the snowfall water equivalent to total precipitation (Huntington et al. 2004; Knowles et al. 2006). In Toronto, correlations with temperature are stronger with the SDF than they are with the ratio between the snowfall equivalent and total precipitation (Hewer and Gough 2020), which may aid in modelling efforts for predicting future changes in the winter season precipitation phase under climate change (McAfee et al. 2014; McCabe et al. 2018). Total freezing days demonstrated greater explanatory power than average winter temperatures for predicting snow-day fractions, which, if combined with other atmospheric variables (Harder and Pomeroy 2013; Marks et al. 2013; Froidurot et al. 2014), may contribute positively to overcoming difficulties modelling precipitation phase. Finally, summing the frequency of rain days, snow days, wet days and freezing days over the winter season produced times series than were normally distributed and less subject to issues of autocorrelation, which is helpful for historical trend detection and future model projections.

## Methods

**Fig. 1:**
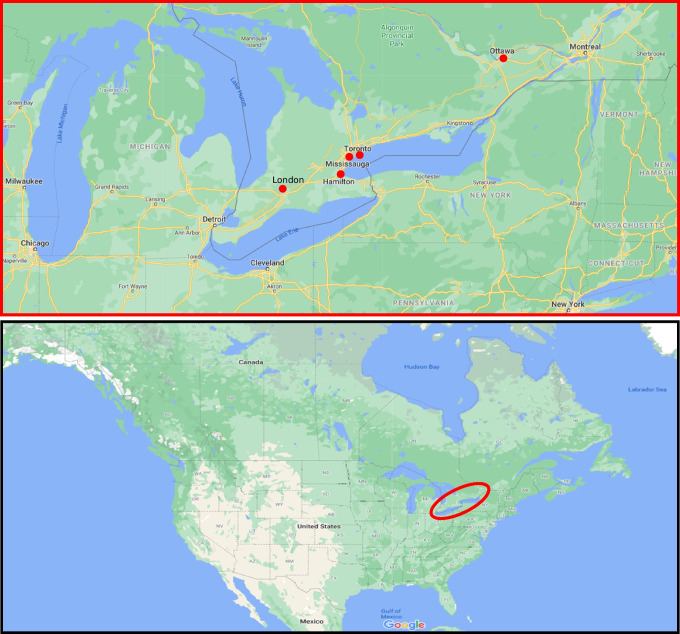
Map of southern Ontario (Canada) showing the 5 study locations (red dots): inset onto a map of North America

Approaching this study from a population geography perspective (Bailey 2011), endeavouring to view climatic change from the lens of lived experience (Renouf 2021), we have considered the most populated cities in southern Ontario. These cities must also have an Environment Canada weather station with approximately 50 years of daily data including average temperature, total rain, total snow and total precipitation. According to the 2016 population census from Statistics Canada, Toronto (Fig. 1) is the most populated city in Ontario with 2,731,571 people and has an Environment Canada weather station located on the University of Toronto downtown campus with the necessary data from 1971 to 2017. Ottawa is the next most populated city in Ontario, with 934,243 people (Statistics Canada 2016), and has a weather station located in the Central Park Experimental Farm with data from 1971 to 2020. Mississauga is the third most populated city in Ontario, with 721,599 people in 2016, and has a weather station with sufficient data located at the Toronto (Lester B. Pearson) International Airport. Although Brampton is officially the 4th most populated city in Ontario (593,638 people in 2016), it was not included in this analysis because of its proximity to Mississauga, where the Toronto International Airport is the closest weather station with sufficient data to both city centres (Pearson is located northeast of Mississauga and southwest of Brampton). Hamilton is the next most populated city in Ontario, with 536,917 people in 2016, and has a weather station located at the Hamilton International Airport with the necessary data from 1971 to 2020. Finally, London will be included as the 5th most populated city in Ontario (383,822 people in 2016), with a weather station located at the London International Airport that recorded the required climate data from 1971 to 2017. These five cities represent approximately 40% of the province’s human population (13.4 million in 2016) while also capturing a range of the southern region’s physical geography spanning from London in the southwest to Ottawa in the northeast, with other central locations situated around Lake Ontario (Hamilton to Toronto).

Daily temperature data (minimum temperature, maximum temperature and mean temperature) as well as daily precipitation data (total rain, total snow and total precipitation) were retrieved from Environment and Climate Change Canada’s historical climate archives for the weather stations located within each of the five sites selected. Initially, we sought to retrieve data for two climate normals: 1961 − 1990 and 1991 − 2020; however, few stations in Ontario consistently record snow and rain data before 1971. Instead, we identified locations that had 50 years of climate data: 1971 − 2020. The five most populated cities in Ontario (excluding Brampton) each had a weather station with the necessary temperature and precipitation data for this recent 50-year period, apart from the London and Toronto stations, which stopped recording rain and snow totals after 2017.

Climate models (Taylor et al. 2012) and climate modelling exercises (Hewer et al. 2016) typically define the winter season as December to February; however, since this study is concerned with the winter season precipitation phase and the occurrence of snow, we have extended the winter season to include March, since snow is common during this month in Ontario. Furthermore, we have employed the continuous winter season approach (Gough et al. 2014; Anderson and Gough 2017), which describes winters in the way people experience them, beginning in December and ending in March of the following year. Therefore, in this study, the winter of 1971 began on December 1st, 1970, and ended on March 31st, 1971; likewise, the winter of 2020 began in December of 2019 and ended in March of 2020.

Since this study is not concerned with the volume but rather the frequency of either rain or snow, recorded measurements were converted into binary codes representing daily occurrences. When analysing the number of rain days, snow days, and wet days across Canada from 1948 − 2016, Vincent et al. (2018) only counted days when precipitation totals exceeded 1 mm, citing inconsistencies in the way trace amounts are recorded over time and between provinces. Since this study covers a shorter and more recent period while only including stations from within southern Ontario, we have elected to count all days with total precipitation greater than or equal to 0.2 mm (trace amounts). When counting the number of rain days and snow days, and then calculating SDF, McAfee et al. (2014) excluded all days with mixed precipitation. In this study, we first omitted trace amounts (< 1 mm) from days with mixed precipitation (following the approach of Vincent et al. 2018), to preserve the effect of days dominated by either rain or snow, then excluded the remaining mixed precipitation days (representing approximately 1.5% of each data set across locations). Additionally, we set snowfall amounts to zero when total precipitation on that day was zero, assuming this was a measurement error associated with blowing snow, since there were trace amounts of snow recorded, despite no precipitation being recorded on that day by Environment Canada. Finally, we confirmed that the total number of wet days recorded each winter season was equal to the number of rain days plus the number of snow days, to ensure an accurate calculation of the snow day fraction (total snow days divided by total wet days).

The main point of analysis in this study is trend detection for the snow day fraction (SDF); however, to better understand changes in SDF, we also analysed trends in rain days, snow days and wet days across each location. Time series trends were described using linear regression (Hewer et al. 2016; Hewer and Gough, 2016, 2020). To validate the assumptions associated with linear regression, we used the Chi-square goodness-of-fit test to determine if the data fit a normal distribution. If the data did not fit a normal distribution, we then considered the results of Kendall’s Tau, a nonparametric rank-order correlation that relaxes the assumption of normality. Furthermore, we used the Durbin-Watson residual test to determine if there were issues of autocorrelation within the data. If statistically significant autocorrelation was detected, we then considered the results of Spearman’s Rho, a monotonic trend analysis that is less susceptible to issues of autocorrelation. Generally, we considered results to be statistically significant if the P value associated with the regression analysis was less than 0.05 (95% confidence level). However, we also reported statistically significant results at the 90% confidence level (*P* < 0.1) while considering the significance of all three statistical correlations (Pearson’s R, Kendall’s Tau and Spearman’s Rho). We also employed another layer of analysis to detect climatic changes where we described differences in means between the first decade (1971 − 1980) and the last decade (2011 − 2020) within the time series. We employed Student’s *t*-tests to determine if the observed differences in mean values between these two decades were statistically significant, using the 90 to 95% confidence intervals. For Toronto and London, the last decade in the time series was 2008 − 2017 since these stations stopped recording the necessary precipitation data after 2017.

Finally, we explored the relationship between warming temperatures and changes in the SDF using Pearson’s coefficient of determination (*R*^2^), Kendall’s Tau (*T*) and Spearman’s Rho (*p*). In addition to exploring to what degree average winter temperatures can explain the observed variability in the SDF, we also considered a new climatic variable we have referred to as freezing days (*T*_mean_ < 0 °C). Environment Canada (2021) defines “frost days” as days when minimum temperatures are below 0 °C, while “freezing degree-days” represent the accumulation of average daily temperatures below 0 °C. Whereas “freezing days” in this study represent the number of days when average temperatures were below 0 °C. This added a temperature-based variable that could similarly be counted and summed over the winter season (December 1 to March 31). Historical trends for freezing days were also detected, and correlations with SDF were explored.

## Results

The city of Toronto is the most populated city in the province of Ontario (as well as within Canada). It is in a central location relative to the more southern locations of Hamilton and London, and the more northern location of Ottawa (Table 1). Due to Toronto’s faster pace of urbanisation and the resulting urban heat island effect (Mohsin and Gough, 2012; Gough 2021), winters in Toronto are already much warmer (by approximately 2 °C) than the other four cities selected across southern Ontario. Toronto recorded the fewest freezing days each winter, as well as the fewest snow days, and has the lowest snow day fraction. Ottawa is the next most populated city; however, we would argue that Mississauga and Brampton combined have more people than Ottawa, and that the area around the Lester B. Pearson International Airport is much more urbanised than the Central Park Experimental Farm location in Ottawa. Regardless due to Ottawa’s more northern location (approximately 250 km north of London), winters are about 3 °C cooler than winters in Mississauga, Hamilton and London. Apart from the warmest, most urbanised city of Toronto and the coolest, most northern city of Ottawa, the remaining three cities had similar temperatures and freezing days, but nuanced differences in snow days and snow day fractions, which will be further explored below.

**Table 1:** Summary statistics for the five most populated cities in Ontario (Canada): including population, weather station location, elevation, and average winter climate conditions (1971 to 2020)

City (station)	Pop. (2016)	Lat., long	Elev. (m)	Temp. (°C)	Freezing days	Rain days	Snow days	Wet days	Snow-day fraction
Toronto (urban campus)	2,731,571	43.67, − 79.40	112.5	-1.53	68	21	28	50	0.56
Mississauga (urban airport)	721,599	43.68, − 79.63	173.4	-3.20	81	21	31	53	0.59
Hamilton (rural airport)	536,917	43.17, − 79.93	237.7	-3.26	83	20	38	58	0.65
London (peri-urban airport)	383,822	43.03, − 81.15	278.0	-3.49	85	20	45	65	0.69
Ottawa (urban park)	934,243	45.38, − 75.72	79.2	-6.58	97	13	40	53	0.75

^*^Population of Ontario (Statistics Canada 2016): 13,448,494

### City of Toronto

**Fig. 2:**
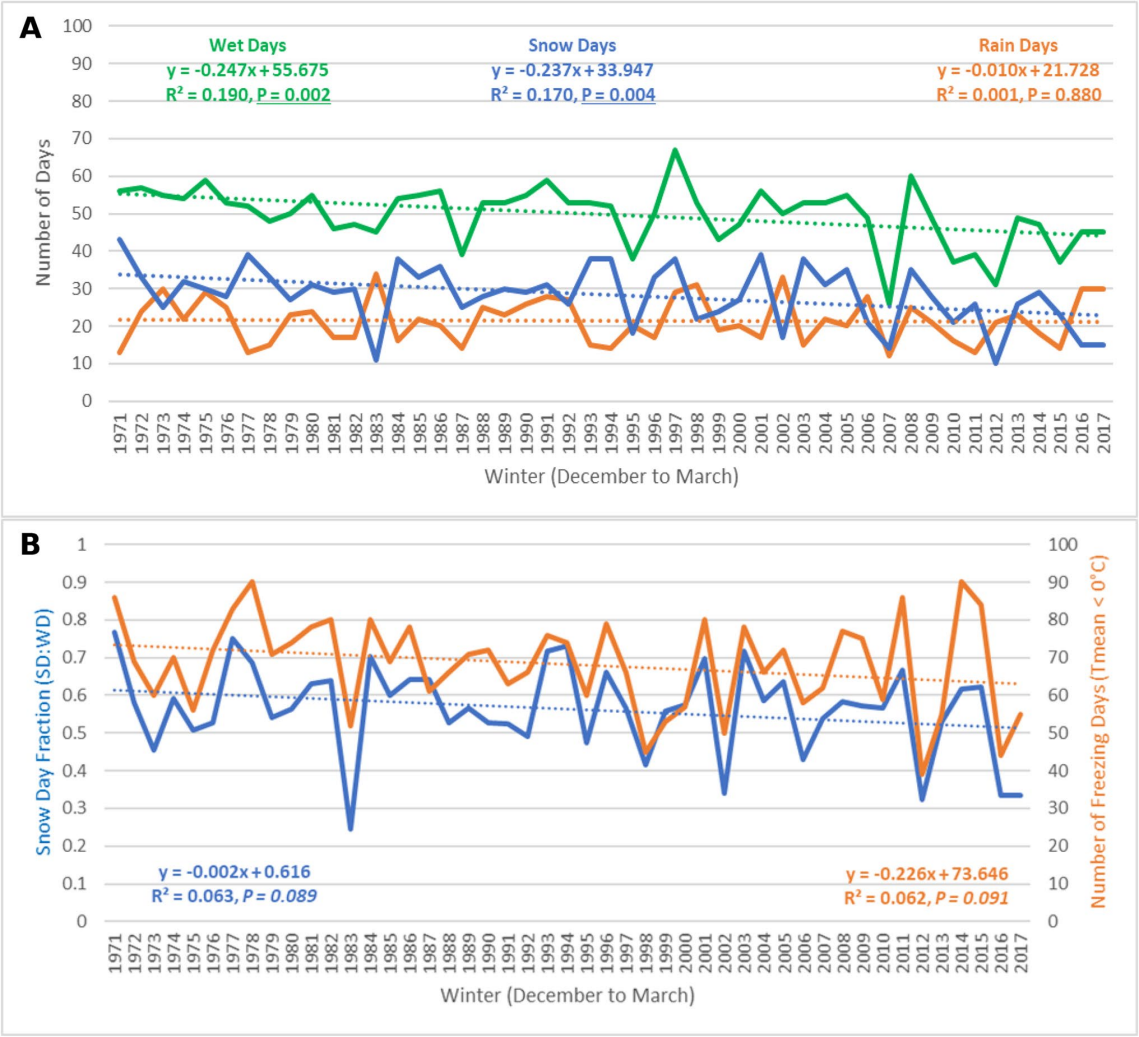
Precipitation- and temperature-based trends for the city of Toronto (University of Toronto) from 1971 to 2017

The total number of winter season wet days (Fig. 2A), recorded at the University of Toronto weather station, has been decreasing at a rate of 1 day every 4 winters (− 0.25 days/year). This trend suggests that average winters in Toronto now experience 12 fewer wet days each season, compared to 50 years ago. This negative linear trend was statistically significant at the 99% confidence level (*R*^2^ = 0.190, *P* = 0.002), and there was no significant autocorrelation detected (*d* = 1.974, *P* > 0.05). Although the data did not fit a normal distribution (*X*^2^ = 4.482, *P* = 0.034), the use of Kendall’s Tau (*T* =  − 0.341, *P* < 0.05), a nonparametric test, validated the statistical significance of Pearson’s *R*. Snow days were also decreasing at a statistically significant rate of approximately 1 less snow day every 4 winters (*R*^2^ = 0.170, *P* = 0.004). Snow days were normally distributed (*X*^2^ = 0.729, *P* = 0.866), and no significant autocorrelation was detected (*d* = 2.250, *P* > 0.05). Total rain days were not associated with a statistically significant trend over the study period, with an average of 21 rain days each winter in Toronto.

The winter season SDF in Toronto has been decreasing at a rate of 0.2% per year from 1971 to 2017 (Fig. 2B), a negative linear trend that was statistically significant at the 90% confidence level (*R*^2^ = 0.063, *P* = 0.089). SDF in Toronto fit a normal distribution (*X*^2^ = 2.709, *P* = 0.100), and no significant autocorrelation was detected (*d* = 2.253, *P* > 0.05). Since both wet days and snow days have been decreasing at a similar rate in Toronto, while the number of rain days remained constant, the negative SDF trend is not as pronounced as it was for snow days alone. Nonetheless, the slope of the linear trend suggests that SDF has decreased by an average of 9% over the study period. From 1971 to 1980, the average SDF in Toronto was 60%, while from 2018 to 2017, the average SDF decreased to 51%, a difference that was statistically significant at the 90% confidence level (*t* = 1.543, *P* = 0.070). The number of days with average temperatures below freezing (0 °C) recorded each winter season in Toronto has also been decreasing at a rate of approximately 1 less freezing day every 4 winters (− 0.23 days/year). This negative linear trend was statistically significant at the 90% confidence level (*R*^2^ = 0.062, *P* = 0.091). Freezing days fit a normal distribution (*X*^2^ = 5.044, *P* = 0.283), and no significant autocorrelation was detected (*d* = 2.153, *P* > 0.05). From 1971 to 1980, Toronto recorded an average of 73 freezing days each winter, while from 2018 to 2017, the average decreased to 66 freezing days; however, this difference was not statistically significant (*t* = 0.999, *P* = 0.166).

Average winter temperatures in Toronto were − 1.53 °C and were increasing at a statistically significant rate of 0.03 °C per year from 1971 to 2020 (*R*^2^ = 0.083, *P* = 0.049). Average winter temperatures were able to explain 62% of the observed variability in Toronto’s SDF during this time, while total freezing days explained 68% of the observed variability in Toronto’s snow day fraction. Although the warming trend detected was stronger with average winter temperatures compared to total freezing days, the predictive power of freezing days was greater in relation to explaining changes in SDF.

### City of Mississauga

**Fig. 3:**
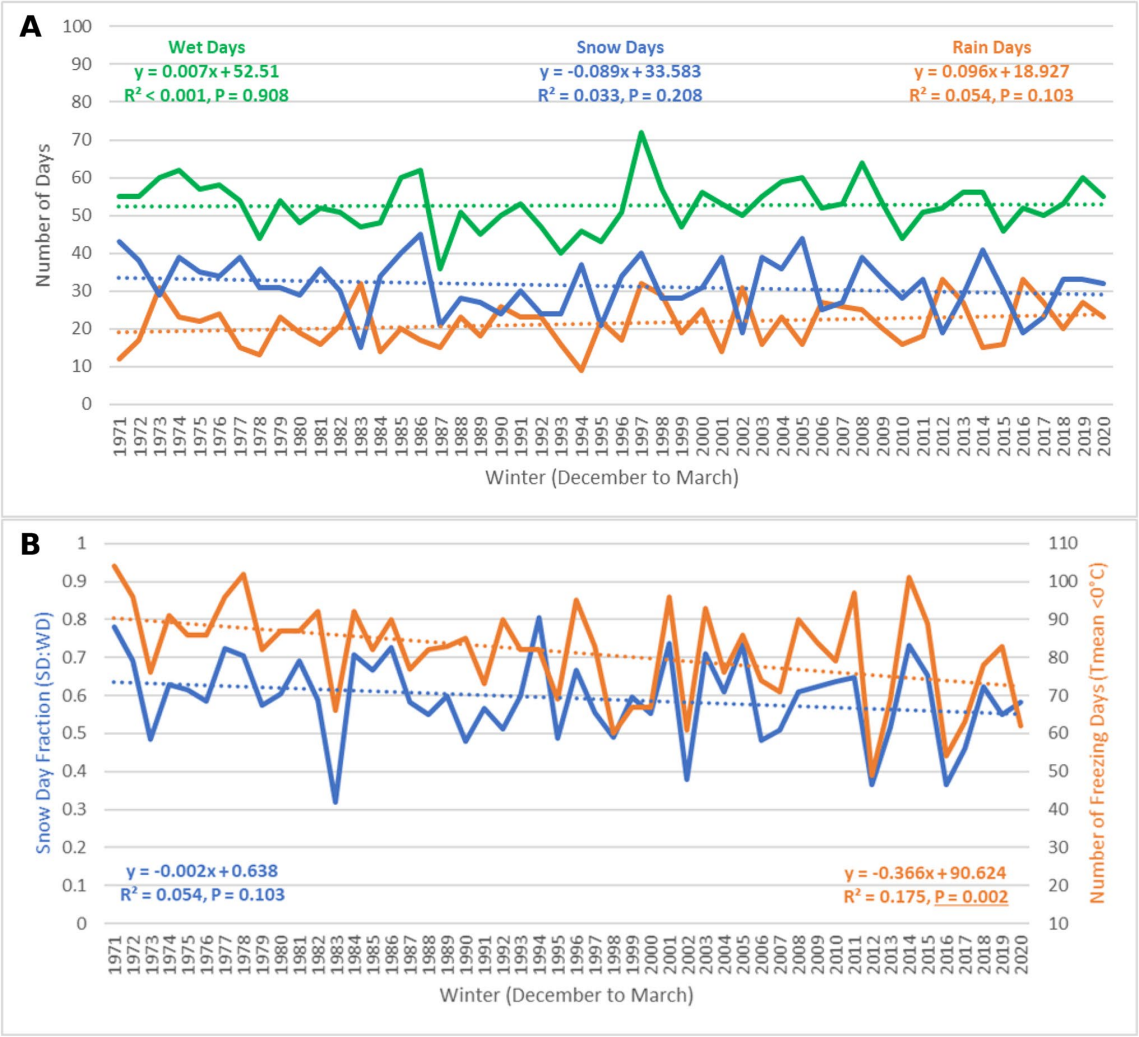
Precipitation- and temperature-based trends for the city of Mississauga (Pearson International Airport) from 1971 to 2020

The total number of wet days each winter season in the city of Mississauga ranged from a low of 36 days in 1987 to a high of 72 days in 1997, with an average of 53 days from 1971 to 2020 (Fig. 3A). The was no indication of the change in total wet days each winter season over this study period. Total snow days from December to March were decreasing at an approximate rate of 1 less snow day every 11 winter seasons (− 0.09 days/year), but this negative trend was not statistically significant (*R*^2^ = 0.033, *P* = 0.208). Snow days in Mississauga fit a normal distribution (*X*^2^ = 4.296, *P* = 0.367), and no significant autocorrelation was detected (*d* = 2.014, *P* > 0.05). Total rain days were increasing at an approximate rate of 1 additional rain day every 10 winter seasons (0.096 days/year), but again, the linear trend was not statistically significant (*R*^2^ = 0.054, *P* = 0.103). Rain days fit a normal distribution (*X*^2^ = 6.022, *P* = 0.198), and no significant autocorrelation was detected (*d* = 2.139, *P* > 0.05). However, according to Spearman’s Rho (*p* = 0.245) and Kendall’s Tau (*T* = 0.167), the positive trend in total winter rain days was statistically significant at that 90% confidence level (*P* < 0.10), giving support to the statistical significance of the linear regression results (Pearson’s *r*).

The fraction of total wet days that recorded snowfall (SDF), during the winter season in Mississauga (Fig. 3B), has been decreasing at a rate of 0.2% per year from 1971 to 2020; however, this trend was not statistically significant (*R*^2^ = 0.054, *P* = 0.103). SDF in Mississauga fit a normal distribution (*X*^2^ = 2.221, *P* = 0.528), and no significant autocorrelation was detected (*d* = 2.288, *P* > 0.05). Neither Spearman’s Rho (*p* =  − 0.211) nor Kendall’s Tau (*T* =  − 0.145) was able to detect a statistically significant time series trend at the 90% confidence level (*P* < 0.10). However, the average SDF in Mississauga from 1971 to 1980 was 64%, while the average SDF decreased to 55% from 2011 to 2020, a difference that was statistically significant at the 95% confidence level (*t* = 1.881, *P* = 0.038). The total number of days when average surface temperatures were below freezing each winter season from 1971 to 2020 has been decreasing at an approximate rate of 1 less freezing day every 3 winter seasons (− 0.37 days/year). This linear trend was statistically significant at the 95% confidence level (*R*^2^ = 0.175, *P* = 0.002). Total freezing days in Mississauga fit a normal distribution (*X*^2^ = 2.893, *P* = 0.576), and no significant autocorrelation was detected (*d* = 2.333, *P* > 0.05). From 1971 to 1980, winters in Mississauga had an average of 91 freezing days, while winters from 2011 to 2020 had an average of 76 freezing days, a difference that was statistically significant (*t* = 2.545, *P* = 0.010).

Average winter temperatures in Mississauga from 1971 to 2020 were − 3.2 °C and were increasing at a statistically significant rate of 0.06 °C per year (*R*^2^ = 0.215, *P* = 0.001). Average winter temperatures were able to explain 61% of the observed variability in the SDF during this time series, while total freezing days explained 66% of the observed variability in Mississauga’s snow day fraction. Although the warming trend detected was stronger for average winter temperatures compared to total freezing days, the predictive power of freezing days was once again greater in relation to explaining changes in SDF.

### City of Hamilton

**Fig. 4:**
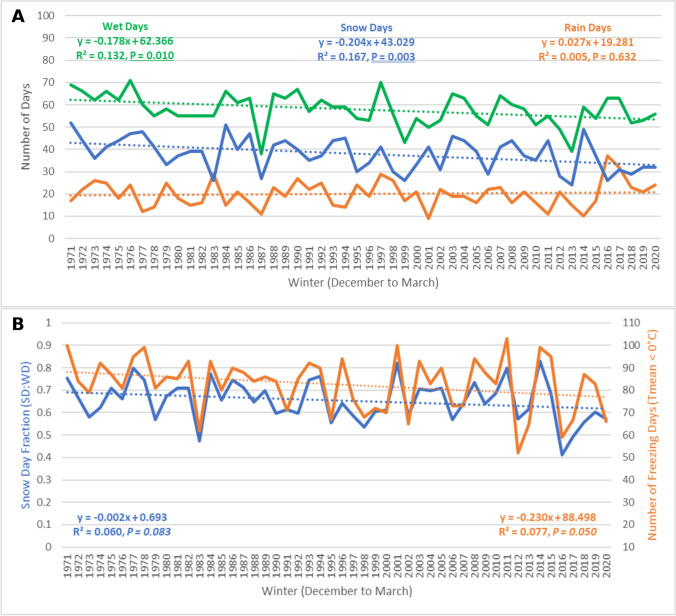
Precipitation- and temperature-based trends for the city of Hamilton (Hamilton International Airport) from 1971 to 2020

The total number of wet days recorded at the Hamilton International Airport each winter from 1971 to 2020 (Fig. 4A) has been decreasing at a statistically significant rate (*R*^2^ = 0.132, *P* = 0.010) of approximately 1 less wet day every 6 winters (− 0.18 days/year). Wet days in Hamilton fit a normal distribution (*X*^2^ = 7.194, *P* = 0.207), and no significant issue of autocorrelation was detected (*d* = 1.865, *P* > 0.05). Snow days have been decreasing at an even faster rate of 1 less snow day every 5 winters (− 0.20 days/year). This negative linear trend was statistically significant at the 99% confidence level (*R*^2^ = 0.167, *P* = 0.003). Snow days fit a normal distribution (*X*^2^ = 4.534, *P* = 0.339), and no significant autocorrelation was detected (*d* = 2.075, *P* > 0.05). The total number of rain days recorded each winter season in the city of Hamilton ranged from a low of 9 days in 2001 to a high of 37 days in 2016, with an average of 20 winter rain days from 1971 to 2020. The was no clear indication of the change in the number of rain days in Hamilton over the study period.

Winter season SDF in Hamilton from 1971 to 2020 (Fig. 4B) has been decreasing at a rate of 0.2% per year for a total reduction in SDF of − 10% over the 50-year study period. This negative linear trend was statistically significant at the 90% confidence level (*R*^2^ = 0.060, *P* = 0.083). SDF in Hamilton fit a normal distribution (*X*^2^ = 3.601, *P* = 0.308), and no significant autocorrelation was detected (*d* = 1.943, *P* > 0.05). Kendall’s Tau (*T* =  − 0.184) and Spearman’s Rho (*p* =  − 0.261) also indicated that the time series trend was statistically significant at the 90% confidence level (*P* < 0.10). From 1971 to 1980, SDF in Hamilton was, on average, 68%, while from 2011 to 2020, SDF decreased to an average of 61%. This difference in SDF between the first decade and the last decade in the time series was also statistically significant at the 90% confidence level, according to Student’s *t*-test (*t* = 1.359, *P* = 0.095). The total number of days when average temperatures were below freezing (0 °C) each winter season has been decreasing in Hamilton at an approximate rate of 1 less freezing day every 4 winters (− 0.23 days/year). This negative linear trend was statistically significant (*R*^2^ = 0.077, *P* = 0.050). Freezing days in Hamilton fit a normal distribution (*X*^2^ = 7.393, *P* = 0.117), and no statistically significant issue of autocorrelation was detected (*d* = 2.394, *P* > 0.05). From 1971 to 1980, there were an average of 88 freezing days in Hamilton each winter season, while from 2011 to 2020, the average number of freezing days decreased to 78, a difference that was statistically significant at the 95% confidence level (*t* = 1.742, *P* = 0.049).

Average winter temperatures in Hamilton were − 3.26 °C and were increasing at a statistically significant rate (90% confidence level) of 0.03 °C per year from 1971 to 2020 (*R*^2^ = 0.060, *P* = 0.087). Average winter temperatures were able to explain 57% of the observed variability in the SDF during this time series, while total freezing days explained 71% of the observed variability in Hamilton’s snow day fraction. In the case of Hamilton, total freezing days were able to better detect the warming trend and had greater predictive power in relation to SDF when compared to average winter temperatures.

### City of London

**Fig. 5:**
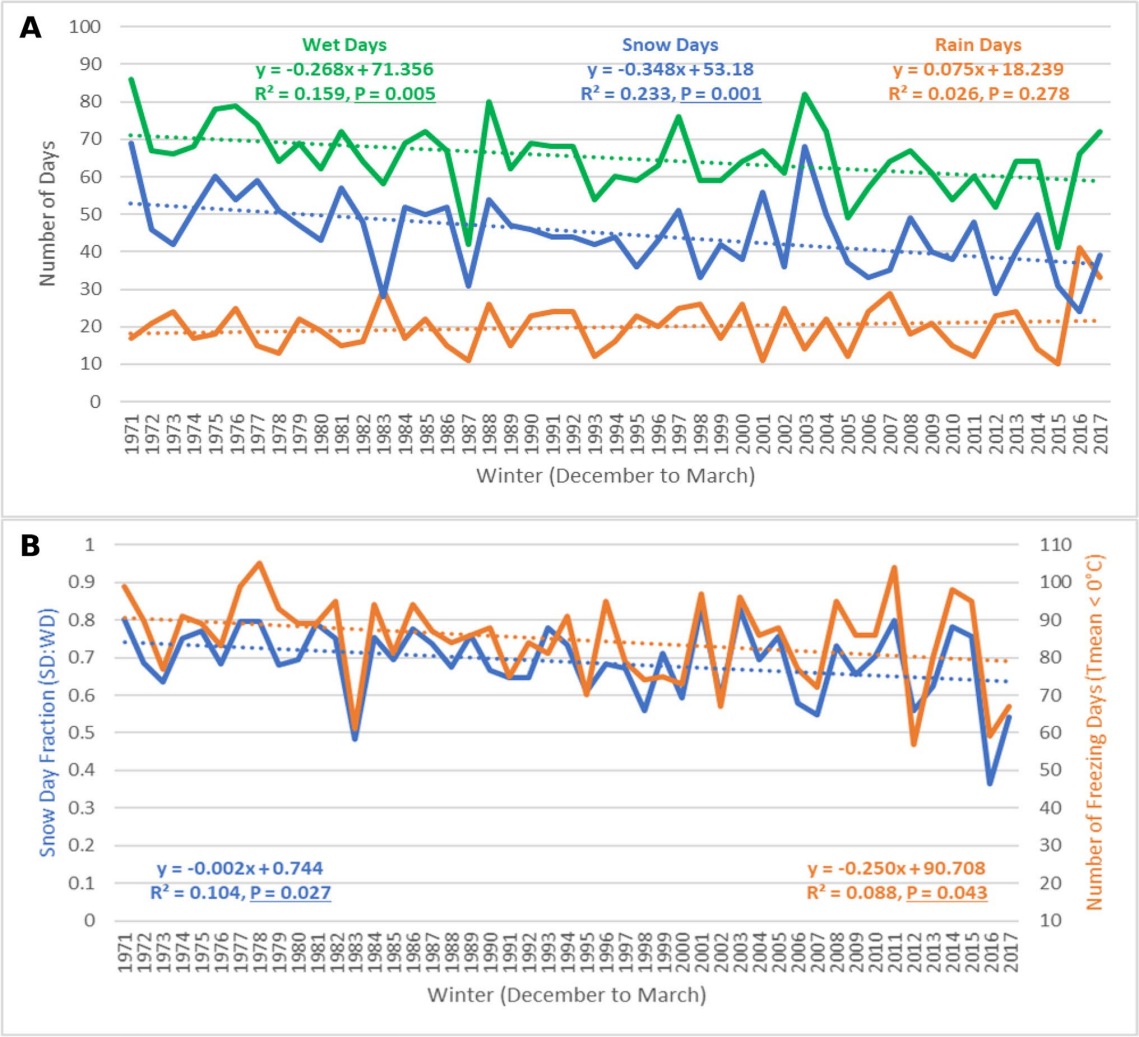
Temperature- and precipitation-based trends for the city of London (London International Airport) from 1971 to 2017

The average number of wet days (excluding mixed precipitation days) in the city of London each winter season from 1971 to 2017 was 65 days, with an average of 45 snow days and 20 rain days. The total number of wet days each winter (Fig. 5A) has been decreasing at an approximate rate of 1 less wet day every 4 winters (− 0.27 days/year). This negative linear trend was statistically significant at the 99% confidence level (*R*^2^ = 0.159, *P* = 0.005). Wet days in London fit a normal distribution (*X*^2^ = 1.525, *P* = 0.677), and no significant issue of autocorrelation was detected (*d* = 2.111, *P* > 0.05). Snow days have also been decreasing in London, at an even greater rate of approximately 1 less snow day every 3 winters (− 0.35 days/year). This negative linear trend was statistically significant at the 99% confidence level (*R*^2^ = 0.233, *p* = 0.001), the data fit a normal distribution (*X*^2^ = 0.545, *P* = 0.909), and no significant autocorrelation was detected (*d* = 2.207, *P* > 0.05). Although there was a slight indication that the total number of rain days recorded each winter season may have been increasing in London over this study period (approximately 1 more rain day every 13 winters), the linear trend was not statistically significant (*R*^2^ = 0.026, *P* = 0.278).

In London, the percentage of wet days that recorded snowfall (SDF) each winter from 1971 to 2017 has been decreasing at a statistically significant rate of 0.2% per year (Fig. 5B). This negative linear trend associated with the SDF in London was statistically significant at the 95% confidence level (*R*^2^ = 0.104, *P* = 0.027), while the data fit a normal distribution (*X*^2^ = 2.252, *P* = 0.522), and there was no significant autocorrelation detected (*d* = 2.284, *P* > 0.05). From 1971 to 1980, the average SDF in London was 73%, while from 2008 to 2017, the average SDF decreased to 65%, a difference that was statistically significant at the 90% confidence level (*t* = 1.673, *P* = 0.056). The total number of days when average surface temperatures were below 0 °C each winter season in London was also decreasing from 1971 to 2017, at a rate of 1 less freezing day every 4 winters (− 0.25 days/year). This negative linear trend was statistically significant at the 95% confidence level (*R*^2^ = 0.088, *P* = 0.043), while the data fit a normal distribution (*X*^2^ = 5.059, *P* = 0.168), and there was no significant autocorrelation detected (*d* = 2.363, *P* > 0.05). During the first decade within the time series, there was an average of 92 freezing days each winter in London, while during the last decade in the study period, the average decreased to 83 days, a difference that was statistically significant at 90% confidence level (*t* = 1.504, *P* = 0.075).

Average winter temperatures in London were − 3.49 °C and were increasing at a statistically significant rate (90% confidence level) of 0.04 °C per year from 1971 to 2017 (*R*^2^ = 0.075, *P* = 0.062). Average winter temperatures were able to explain 61% of the observed variability in during this time series, while total freezing days explained 75% of the observed variability in London’s snow day fraction. For London, total freezing days were able to better detect the warming trend and had greater predictive power in relation to SDF when compared to average winter temperatures.

### City of Ottawa

**Fig. 6:**
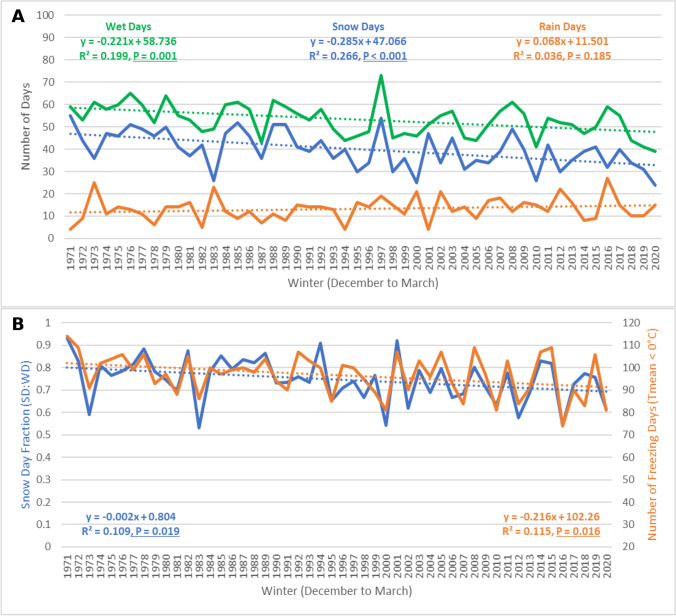
Temperature- and precipitation-based trends for the city of Ottawa (Central Park Experimental Farm) from 1971 to 2020

The total number of wet days (excluding mixed precipitation) during the winter season (December to March) in the city of Ottawa (Fig. 6A) has been decreasing at a statistically significant rate (*R*^2^ = 0.199, *P* = 0.001) of 1 less wet day every 4.5 winters (− 0.22 days/year). Total snow days were decreasing at an even faster rate of 1 less snow day every 3.5 winter seasons (− 0.29 days/year). This negative linear trend was statistically significant (*R*^2^ = 0.266, *P* < 0.001), the data fit a normal distribution (*X*^2^ = 1.893, *P* = 0.755), and no significant autocorrelation was detected (*d* = 2.177, *P* > 0.05). Total rain days were increasing at an approximate rate of 1 more rain day every 15 winter seasons (0.07 days/year), but this linear trend was not statistically significant (*R*^2^ = 0.036, *P* = 0.185). Rain days in Ottawa fit a normal distribution (*X*^2^ = 4.391, *P* = 0.356). There was evidence of positive serial correlation (*d* = 2.485, *P* < 0.05), which may have confounded Pearson’s *r*, potentially producing a false negative. However, both Spearman’s Rho (*p* = 0.213) and Kendall’s Tau (*T* = 0.155) also concluded that this positive trend in total rain days was not statistically significant at the 90% confidence level (*P* > 0.10).

The fraction of total wet days that recorded snowfall (SDF) during the winter season in Ottawa has been decreasing at a statistically significant rate (*R*^2^ = 0.109, *P* = 0.019) of 0.2% per year from 1971 to 2020 (Fig. 6B). SDF in Mississauga fit a normal distribution (*X*^2^ = 1.048, *P* = 0.790), and no significant autocorrelation was detected (*d* = 1.825, *P* > 0.05). From 1971 to 1980, the average SDF in Ottawa was 79%, while the average SDF decreased to 71% from 2011 to 2020, a difference that was statistically significant at the 95% confidence level (*t* = 1.944, *P* = 0.034). The total number of days when average surface temperatures were below freezing each winter season from 1971 to 2020 has been decreasing at an approximate rate of 1 less freezing day every 4.5 winter seasons (− 0.22 days/year). This negative linear trend was statistically significant at the 95% confidence level (*R*^2^ = 0.115, *P* = 0.016). Total freezing days in Ottawa fit a normal distribution (*X*^2^ = 3.358, *P* = 0.340), but there was evidence of negative serial correlation (*d* = 2.464, *P* < 0.10); however, Spearman’s monotonic trend detection confirmed the statistical significance of this negative time series trend at the 90% confidence level (*p* =  − 2.464). From 1971 to 1980, Ottawa recorded an average of 102 freezing days each winter, while from 2011 to 2020, winters in Ottawa had an average of 93 freezing days, a difference that was statistically significant (*t* = 2.032, *P* = 0.029).

Average winter temperatures in Ottawa were − 6.6 °C and were increasing at a statistically significant rate of 0.05 °C per year from 1971 to 2020 (*R*^2^ = 0.140, *P* = 0.003). Average winter temperatures were able to explain 59% of the observed variability in the SDF from 1971 to 2020 in Ottawa, while total freezing days explained 67% of the observed variability in Ottawa’s snow day fraction. Although the local warming trend was stronger with average winter temperatures compared to total freezing days, the predictive power of freezing days was greater than surface temperatures in relation to explaining changes in SDF.

## Intercity comparison and discussion

Looking at the slope of the linear trend line for SDF from 1971 to 2020 (Table 2), London and Ottawa recorded the most pronounced decline (− 0.23% and − 0.22% per year, respectively), with the highest statistical significance (99% confidence level). Hamilton and Mississauga recorded the least pronounced decline in SDF (− 0.15% and − 0.17% per year, respectively), with both trends only being statistically significant at the 90% confidence level. Although the negative slope of the linear trend line was quite pronounced for the city of Toronto (− 0.22% per year); unlike London and Ottawa, the results were only statistically significant at the 90% confidence level. When comparing SDF during the first decade of the time series (1971 − 1980) to SDF during the most recent decade (2011 − 2020), Mississauga and Ottawa recorded the greatest differences (− 9% and − 8%, respectively) and the most statistically significant results (95% confidence levels). The remaining three cities recorded similar differences in the range of − 7% (Hamilton) to − 9% (Toronto), but the differences between means were only significant at the 90% confidence level. From these two measures of change in SDF, it can be concluded that the greatest degree of change was observed among the two coldest and snowiest sites: being the most northern location (Ottawa) and the most southern location (London). Although the change was also detected across the other three cities, the effects may have been moderated across these sites due to their closer proximity to Lake Ontario and the warming effects that large water bodies have on winter temperatures. Another explanation is that since the temporal scope of this study is limited to a recent 50-year period (1971 − 2020), these warmer locations may have already experienced considerable climatic change earlier in the twentieth century (e.g., 1951 − 1970).

**Table 2:** Summary of climatic changes in winter season snow day fraction (SDF) and freezing days (FDs) across Ontario’s five most populated cities

City	*m* SDF, 1971 − 2020	μ SDF, 1971 − 1980	μ SDF, 2011 − 2020	∆ SDF	*m* FDs, 1971 − 2020	μ FDs, 1971 − 1980	μ FDs, 2011 − 2020	∆ FDs	SDF* FDs
TOR	*m* = *− 0.22%* *P* = *0.089*	60%	51%	*t* = *1.543* *P* = *0.070*	*m* = *− 0.23 d* *P* = *0.091*	73 d	66 d	t = 0.999 P = 0.166	**R**^**2**^** = 0.675**
MIS	m = − 0.17% P = 0.103	64%	55%	**t = 1.881** **P = 0.038**	**m = − 0.37 d** **P = 0.002**	91 d	75 d	**t = 2.545** **P = 0.010**	**R**^**2**^** = 0.616**
HAM	*m* = *− 0.15%* *P* = *0.083*	68%	61%	*t* = *1.359* *P* = *0.095*	*m* = *− 0.23 d* *P* = *0.050*	88 d	78 d	**t = 1.742 P = 0.049**	**R**^**2**^** = 0.682**
LON	**m = − 0.23%** **P = 0.027**	73%	65%	*t* = *1.673* *P* = *0.056*	**m = − 0.25 d** **P = 0.043**	92 d	83 d	*t* = *1.504 P* = *0.075*	**R**^**2**^** = *0.760***
OTT	**m = − 0.22%** **P = 0.019**	79%	71%	***t* = 1.944 *P* = 0.034**	**m = − 0.22 d** **P = 0.016**	102 d	93 d	**t = 2.032 P = 0.029**	**R**^**2**^** = 0.621**

^*^Bold indicates statistical significance at the 95% confidence level (*P* < 0.05). *Italics* indicates statistical significance at the 90% confidence level (*P* < 0.10). ***m*** is the slope of the linear trend over the 50-year time series. **μ** is the mean over the first and last decades in the times series. **∆** is a measure of climatic change between the beginning and end of the time series using Student *t*-tests (*t*) to determine if the difference in means between the first and last decades were statistically significant

The greatest change in the average number of freezing days (FDs) each winter season was observed in Mississauga, where FDs have been decreasing by − 0.37 days per year at the 95% confidence level. The remaining four cities recorded decreases between − 0.22 and − 0.25 freezing days per winter season, where the negative linear trends for London and Ottawa were statistically significant at the 95% confidence level, while the trends in Toronto and Hamilton were only significant at the 90% confidence level. Mississauga also recorded the greatest difference between FDs during the first decade and FDs during the last decade (− 16 days), a difference that was statistically significant at the 99% confidence level. Hamilton and Ottawa recorded statistically significant differences between these two decades at the 95% confidence level (− 10 FDs and − 9 FDs, respectively), while London recorded a similar difference (− 9 FDs) but was only significant at the 90% confidence level. Toronto recorded the smallest difference in freezing days between decades (− 7 FDs), and the difference was not statistically significant. It is important to acknowledge that Toronto has already experienced 2-degree warming compared to the other three southern locations (Mississauga, Hamilton and London) and that this recent 50-year period may exclude a portion of the warming trend already observed (Mohsin and Gough 2010).

The strongest correlation between SDF and FDs was reported for London, where the total number of freezing days each winter season was able to explain 76% of the observed variability in the snow-day fraction from 1971 to 2017 (*N* = 47). Both SDF and FDs in London were decreasing at statistically significant rates (95% confidence level). The next strongest correlations were reported in Toronto and Hamilton, where changes in FDs were able to explain 68% of the observed variability in SDF across these two cities (*N* = 47 and *N* = 50, respectively). Both the SDF and FDs in Toronto and Hamilton have been decreasing at statistically significant rates, but only at the 90% confidence levels. The weakest correlations between FDs and the SDF were reported for Mississauga and Ottawa, where changes in FDs were able to explain 62% of the observed variability in SDF over the study period (*N* = 50 for both cities). Although Mississauga recorded the strongest decline in FDs, it recorded the second least pronounced decline in SDF, whereas Ottawa recorded one of the greatest rates of decline in both FDs and SDF, yet still had a weaker correlation between these two variables.

**Fig. 7:**
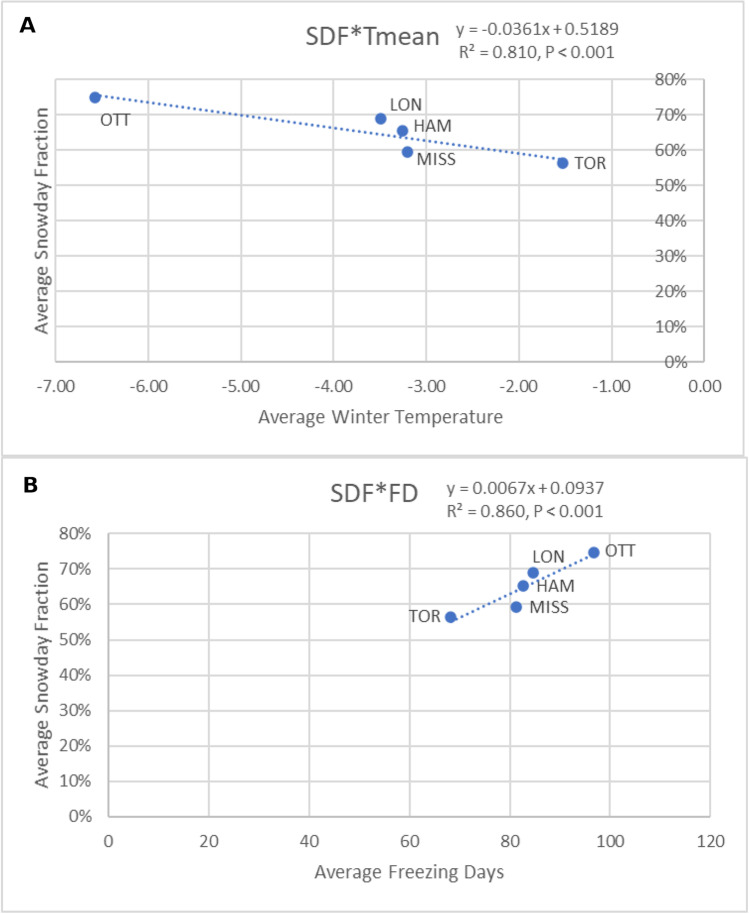
Average snow day fraction as a function of average winter temperature (**a**) and average freezing days (**b**) for the five most populated cities in southern Ontario (Canada) from 1971 to 2020

In a final effort to interpret the results of this study and discuss the difference between cities, Fig. 7 plots average winter season snow day fractions (SDF) for each of the five cities as a function of average winter temperatures (7A) and as a function of the average number of freezing days (7B). Despite the very small sample size (*N* = 5), the average number of freezing days was able to explain 86% of the observed variability in winter season snow day fractions across these five cities. Once again, freezing days had greater explanatory power than average temperatures, demonstrating the utility of this frequency-based temperature threshold exceedance variable for predicting the winter season precipitation phase. Due to its proximity to Lake Ontario and the highest rate of urbanisation, Toronto is by far the warmest city, with the fewest freezing days and the lowest snow day fraction. Since Ottawa is the most northern location and furthest from all the Great Lakes, it is by far the coldest city with the most freezing days and the highest snow day fraction. The Hamilton weather station is situated on the Niagara escarpment, in the rural town of Mount Hope, approximately 20 km south of Lake Ontario; therefore, winter temperatures and freezing days are not being diminished by lake effects or urban heat island effects. Mississauga and London are special cases. Winter temperatures and freezing days in Mississauga are like those in Hamilton, but the SDF in Mississauga is lower than expected (more like Toronto), likely due to similar rates or urbanisation (Anderson et al. 2018) and proximity to the northern shore of Lake Ontario (Anderson and Gough 2017). Finally, winter temperatures and freezing days in London are also like those recorded in Hamilton, yet the SDF in London is higher than expected (more like Ottawa), likely because London is situated in the Great Lakes convergence zone and considered to be part of the Lake Huron Snowbelt (Wiley and Mercer 2021).

## Conclusions

It is difficult to make any direct comparisons with the results of this study and the existing academic literature since few studies have employed the snow-day fraction in such a manner. McAfee et al. (2014) used SDF to describe the precipitation phase across the US state of Alaska but did so on a monthly time scale and were concentrated on using a temperature-based model to predict SDF rather than analysing time series trends. Vincent et al. (2018) analysed trends in rain days, snow days and wet days across Canada, but did not calculate the snow-day fraction. Hewer and Gough (2020) looked at SDF for Toronto from 1849 to 2017 but did not employ the same rigorous data treatments (removing blowing snow errors, removing trace precipitation amounts from mixed precipitation days, and then excluding the remaining mixed precipitation days from the analysis). In general, the results of this study agree with the national consensus that temperatures are warming (Zhang et al. 2000, 2019) while snowfall is decreasing (Vincent et al. 2015, 2018). However, these localised results do not agree with the findings from broader geographic studies, suggesting that the number of rainy days and wet days is increasing in southern Ontario (Vincent and Mekis 2006 Mekis and Vincent 2011).

The current study demonstrates the usefulness of the SDF as a measure of climatic change, and future research should expand the geographic scope of this study to include other locations across Canada as well as other international studies in regions where precipitation falls as snow. The snow-day fraction may also be a useful measure within the field of applied climatology and climate change impact assessment, with potential applications for outdoor recreation and tourism (Hewer and Gough 2018), including winter activities like snowmobiling (McBoyle et al. 2007), downhill skiing (Scott et al. 2019) and cross-country skiing (Hewer et al. 2016). Another potentially useful application of the SDF could involve the transportation sector (Picketts et al. 2016), including road safety (Hambly et al. 2013), pavement maintenance (Tighe et al. 2008) and winter (ice) roads (Hori et al. 2017, 2018).

Snow is a good indicator of climate change since humans can observe changes in snow and ice better than they can recognise gradual changes in temperature. For example, ice coverage over Lake Ontario has decreased considerably over the last century (Hewer and Gough 2019), and these changes are more noticeable to local residents than the warming of air and water temperatures driving them. Snow days and the snow-day fraction are decreasing across southern Ontario. They are decreasing at greater rates northeast of Lake Ontario (Ottawa) as well as southwest of the lake (London). Toronto has already experienced greater warming than the other cities in Ontario (warmest temperatures, fewest freezing days and lowest snow-day fraction) due to its faster pace of urbanisation and resulting heat island effect (Gough 2020, 2021). Toronto and Mississauga may also be subject to lake effects that are moderating winter temperatures (Gough and Rosanov 2001), reducing freezing days and decreasing snow-day fractions. Strong correlations with temperatures and freezing days combined with the statistical properties of the snow-day fraction (normal distribution, no autocorrelation) show the potential to make predictive models more effective for climate change projections, with positive implications for the field of applied climatology and the practice of climate change impact assessment.

## Data Availability

The datasets generated and analysed during the current study are available in the Government of Canada’s historical climate data archive, available at: https://climate.weather.gc.ca/historical_data/search_historic_data_e.html.
